# Optimization of Bromelain-Aided Production of Angiotensin I-Converting Enzyme Inhibitory Hydrolysates from Stone Fish Using Response Surface Methodology

**DOI:** 10.3390/md15040104

**Published:** 2017-03-31

**Authors:** Shehu Muhammad Auwal, Mohammad Zarei, Azizah Abdul-Hamid, Nazamid Saari

**Affiliations:** 1 Department of Food Science, Faculty of Food Science and Technology, University Putra Malaysia, Serdang, Selangor 43400, Malaysia; samuhammad.bch@buk.edu.ng (S.M.A.); mzarei.mail@gmail.com (M.Z.); azizahah@upm.edu.my (A.A.-H.); 2 Department of Biochemistry, Faculty of Basic Medical Sciences, Bayero University, Kano 700231, Nigeria; 3 Department of Food Science and Technology, College of Agriculture and Natural Resources, Sanandaj Branch, Islamic Azad University, Sanandaj 66131, Iran

**Keywords:** ACE inhibitory hydrolysates, degree of hydrolysis, stone fish, central composite design, response surface methodology

## Abstract

The stone fish is an under-utilized sea cucumber with many nutritional and ethno-medicinal values. This study aimed to establish the conditions for its optimum hydrolysis with bromelain to generate angiotensin I-converting enzyme (ACE)-inhibitory hydrolysates. Response surface methodology (RSM) based on a central composite design was used to model and optimize the degree of hydrolysis (DH) and ACE-inhibitory activity. Process conditions including pH (4–7), temperature (40–70 °C), enzyme/substrate (E/S) ratio (0.5%–2%) and time (30–360 min) were used. A pH of 7.0, temperature of 40 °C, E/S ratio of 2% and time of 240 min were determined using a response surface model as the optimum levels to obtain the maximum ACE-inhibitory activity of 84.26% at 44.59% degree of hydrolysis. Hence, RSM can serve as an effective approach in the design of experiments to improve the antihypertensive effect of stone fish hydrolysates, which can thus be used as a value-added ingredient for various applications in the functional foods industries.

## 1. Introduction

Marine invertebrates are naturally endowed with valuable proteins that can be exploited in the production of functional hydrolysates and their constituent’s biopeptides. Many peptides exhibiting one or more biological activities including those for the production of antimicrobial, antioxidant, antithrombotic, anti-Alzheimer, opiate and antihypertensive activity have been reported [1]. The angiotensin I-converting enzyme (ACE) acts as a key enzyme in blood pressure regulation via its dual effects on the renin angiotensin as well as the kinin-kallikrein pathways. The Zn-containing peptidyl dipeptide hydrolase triggers the conversion of Angiotensin I to a potent vasopressor, Angiotensin II [2], and promotes the inactivation of bradykinin, an active vasodilator, resulting in elevated blood pressure or hypertension [3]. The mechanism of the vasopressor effect involves the release of aldosterone, a sodium-retaining steroid from the adrenal cortex [4]. Inhibition of ACE by decreasing angiotensin II formation and increasing bradykinin production is necessary to control elevated blood pressure or hypertension [5,6].

Most commercially-available synthetic ACE inhibitors, such as enalapril, captopril, and lisinopril, have been reported to effectively reduce blood pressure by blocking the formation of angiotensin II from angiotensin I [7]. However, they are often costly and are associated with certain undesirable side effects including skin rashes, coughing and allergic reactions [8].

Therefore, development of food protein hydrolysates with an ACE-inhibitory effect is considered to be a safe alternative to the use of the synthetic ACE inhibitors in the management of hypertension [2].

Protein hydrolysates and peptides with ACE-inhibitory activities are the most studied among the different classes of food biopeptides [9]. They occur as inactive sequences within the primary structure of their parent protein and are released via properly-designed enzymatic hydrolysis. However, most of the ACE-inhibitory hydrolysates derived from marine invertebrates were reported to exhibit low bioactivity that varies with the type of hydrolyzing enzyme being used [10].

Thus, the choice of an appropriate enzyme is critical for obtaining inhibitory hydrolysates with strong activity against ACE. As previously reported, alcalase and bromelain-generated hydrolysates yielded the highest ACE-inhibitory activity when stone fish (*Actinopyga lecanora*) was hydrolyzed by six different proteases whereas the activity decreased with trypsin, papain, pepsin, and flavourzyme generated hydrolysates [10].

The stone fish used in this study is a marine invertebrate that belongs to the phylum echinoderm and class Holothuroidea. It is categorized among the edible species of sea cucumber that are common to Malaysia and other south-Asian sea shores where it is collected by hand picking, free-diving and as by-catch of the fishing industry [11,12]. Despite being abundantly available, easy to propagate and with great commercial potential, the species is still under-utilized. Several biological effects, including antioxidant- [13], antibacterial- and antihypertensive [10,12] effects have been ascribed to stone fish hydrolysates produced using different proteases. *In vitro* and *in vivo* experiments with bromelain-generated hydrolysates of stone fish indicated their significant inhibitory capacity against the angiotensin I-converting enzyme activity [10].

However, the conditions for the hydrolysis of stone fish have not been previously optimized. Hence, the present study applied response surface methodology (RSM) using bromelain to determine the best conditions to produce stone fish hydrolysates with maximum inhibitory effects on ACE. It is hoped that improving the target activity will expand the utilization of *A. lecanora* as a bio resource abundantly available in many countries for various applications in functional foods development.

## 2. Results and Discussion

### 2.1. Analysis of Proximate Composition

The proximate composition analyzed for the powdered stone fish sample is given in Table 1. The moisture, ash, crude fat, crude protein and total carbohydrate contents were 5.11%, 39.80%, 2.03%, 45.39% and 7.68% respectively. The protein content percentage on dry-weight basis was found to be slightly higher than that obtained from other species of sea cucumber (Table 1). Thus, stone fish can serve as a good source of protein for various applications.

### 2.2. Effect of Hydrolysis pH, Temperature, Enzyme/Substrate Ratio and Time on Degree of Hydrolysis and ACE-Inhibitory Activity

The actual and predicted responses for the degree of hydrolysis (DH; Y1) and ACE-inhibitory activity (Y2) of stone fish hydrolysates under the combined effects of the different hydrolysis conditions pH (X1), temperature (X2), E/S ratio (X3) and time (X4) are presented in Table 2. Furthermore, Table 3 shows the analysis of variance at a probability of *p <* 0.05 and estimated regression coefficients for the DH and ACE-inhibitory-activity quadratic models (Y1 and Y2), respectively. Statistical parameters including coefficient of determination (R^2^) and F-test probability have also been indicated. The R^2^ values of Y1 and Y2 were 98.50% and 98.70%, respectively.

#### 2.2.1. Effect of pH, Temperature, E/S Ratio and Time on Degree of Hydrolysis

**Figure 1:**
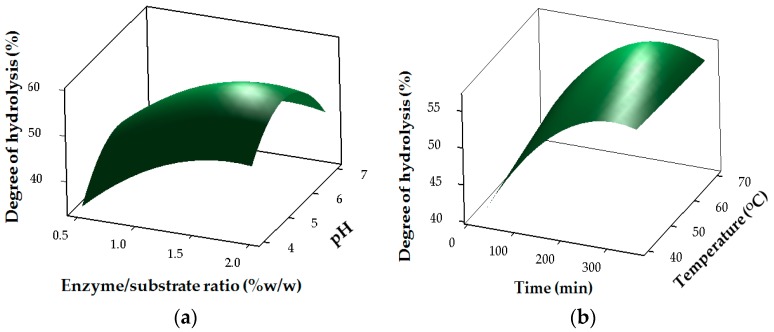

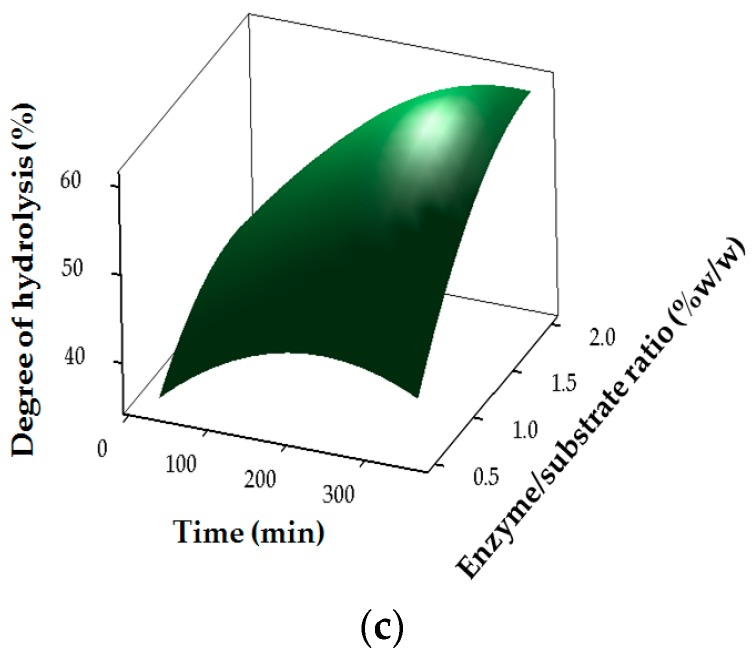
Response surface plots for the effects of independent factors on degree of hydrolysis: (**a**) pH and E/S ratio; (**b**) temperature and time (**c**) enzyme/substrate (E/S) ratio and time.

The results for the degree of hydrolysis of stone fish hydrolysates (SHs) are shown in Table 2 and Figure 1. The regression coefficients revealed the strong linear (*p <* 0.05) and interaction (*p <* 0.05) effects of pH (X1), temperature (X2), E/S ratio (X3) and time (X4) on DH, whereas temperature had no significant (*p <* 0.05) effect in the quadratic region of the model for DH (Table 3). The DH of stone fish hydrolysates increased with the duration of bromelain hydrolysis at an increasing E/S ratio (Figure 1).

Such increase in DH with an increase in hydrolysis time and E/S ratio has been reported previously, [10,16] and was attributed to the maximum cleavage of the polypeptides due to an increased concentration of enzymes acting on the substrate [16,17].

As shown in Figure 3, the theoretical DH value of 46.26% was predicted for the hydrolysis of stone fish protein under the optimum condition of pH (7), temperature (40 °C), E/S ratio (2%) and hydrolysis time (240 min). The experimental DH value of 44.59% obtained under the same hydrolysis condition was not significantly different from the predicted DH value within a 95% confidence interval.

#### 2.2.2. Effect of pH, Temperature, E/S Ratio and Time on ACE-Inhibitory Activity

**Figure 2:**
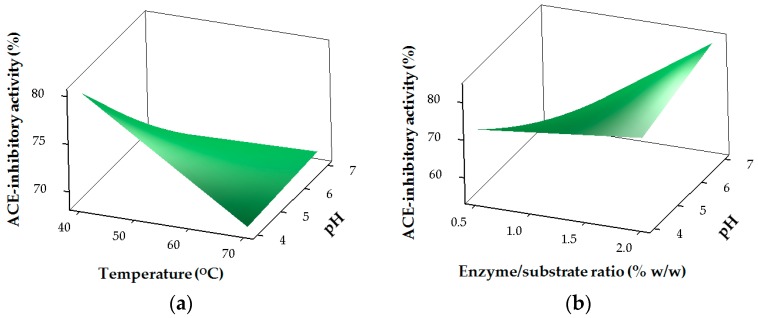

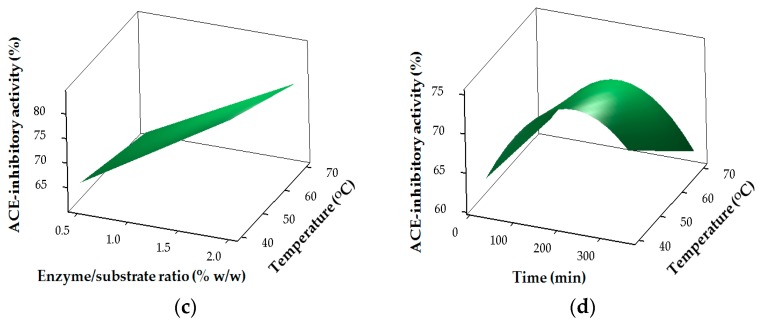
Response surface plots for the effects of the independent factors on angiotensin I-converting enzyme (ACE)-inhibitory activity: (**a**) pH and temperature; (**b**) pH and E/S ratio; (**c**) temperature and E/S ratio (**d**) temperature and time.

The results for the ACE-inhibitory activity of SHs are shown in Table 2 and Figure 2. The regression coefficients revealed the strong linear (*p <* 0.05) and interaction (*p <* 0.05) effects of pH (X1), temperature (X2), E/S (X3) and time (X4) on ACE-inhibitory activity, while only time (X4) had a significant (*p <* 0.05) effect in the quadratic region of the model for ACE-inhibitory activity (Table 3). ACE-inhibitory activity of SHs is greatly influenced by the hydrolysis conditions and the resulting DH. Higher ACE-inhibitory properties for the SHs were mostly obtained at higher DH values (Table 2). Similarly, the type of proteases used and the DH were reported to affect the ACE-inhibitory activity of protein hydrolysates [10,17].

**Figure 3:**
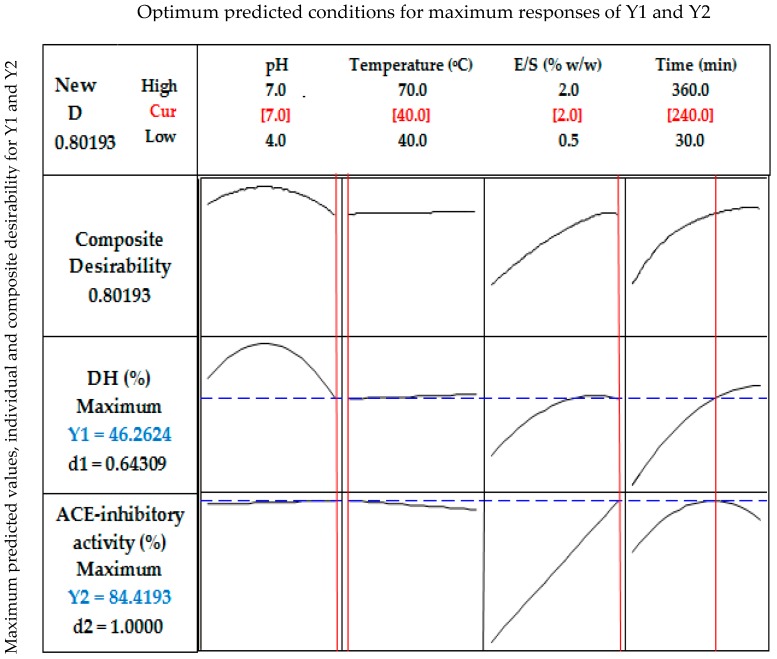
Response optimization for the hydrolysis parameters, predicted responses, and their level of desirability. Y1 = degree of hydrolysis (DH, %), Y2 = ACE-inhibitory activity (%), D = Composite desirability for YI and Y2 responses, d1 = individual desirability of Y1, d2 = individual desirability of Y2. Optimum selected conditions of pH, temperature, enzyme/substrate ratio and time are shown in red whereas the maximum predicted responses of Y1 and Y2 are shown in blue.

The theoretical value of 84.42% for ACE inhibition was predicted (Figure 3) under the optimum hydrolysis conditions for stone fish protein as defined by pH (7), temperature (40 °C), E/S ratio (2%) and hydrolysis time (240 min). The experimental value of 84.26% obtained for ACE inhibition under the same hydrolysis conditions revealed no significant difference compared to its predicted value within a 95% confidence interval.

The strong ACE-inhibitory activity obtained for SHs might be due to the increased E/S ratio under optimum hydrolysis conditions, which resulted in a higher DH and the release of the potent peptides responsible for the observed effects of SHs on ACE.

These results are in agreement with the findings of van der Ven et al. who reported a high ACE-inhibitory effect at a higher DH for whey protein hydrolysates produced with a mixture of pancreatic enzymes [6].

Similarly, the high ACE-inhibitory activity shown by SHs at lower DH can be supported by the work of Guo et al. who reported high ACE inhibition at lower DH for hydrolysates of whey protein concentrate generated using crude proteinases [16].

### 2.3. Functional Properties of Stone Fish Protein Hydrolysates Produced under Optimum Hydrolysis Conditions

The functional properties influence the behaviour of bioactive protein hydrolysates in food, and therefore affect their acceptability by consumers.

Solubility is a desirable functional property and a determinant of the potential applications of proteins and protein hydrolysates in various systems. The degree of solubility of protein is related to the amphiphilic property of its amino acid content, which determines the extent to which it interacts with either water or oil in food system [18].

**Figure 4:**
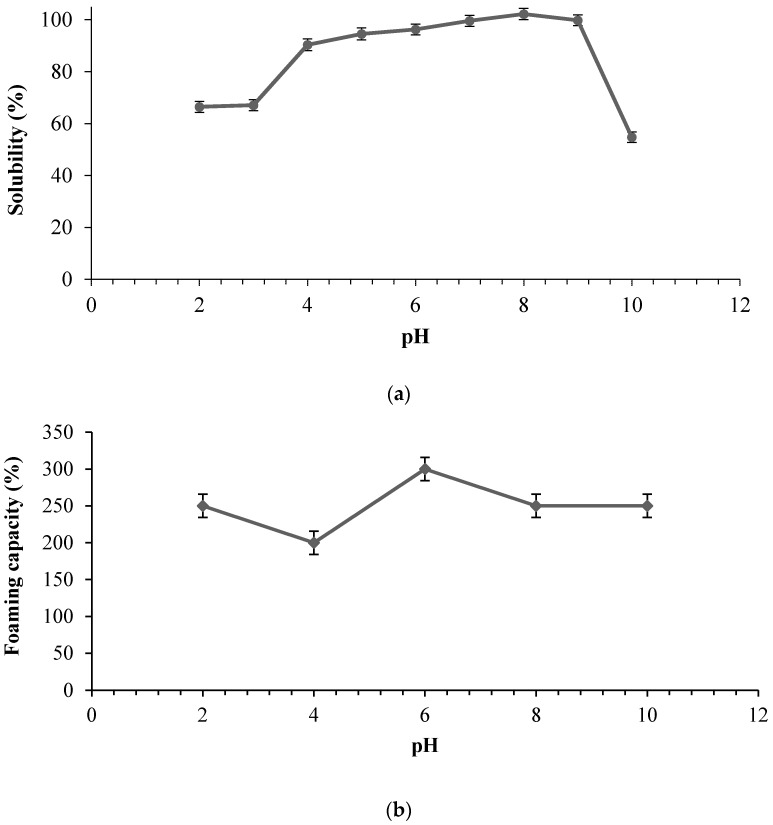

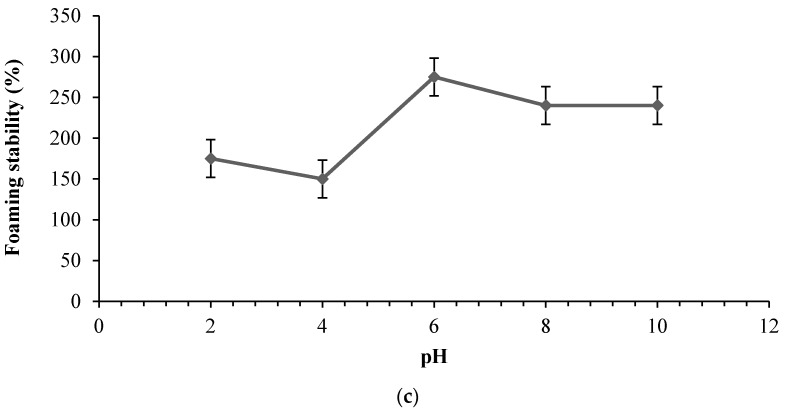
Functional properties of stone fish hydrolysates prepared using bromelain as influenced by pH; (**a**) Solubility profile (**b**) Foaming capacity and (**c**) Foam stability. Each value represents a mean of triplicate determinations.

A significant effect of pH was observed on the solubility of SHs. As shown in Figure 4a, the lowest solubility of SHs was noted at pH 10 while the hydrolysates showed its maximum solubility (almost 100%) between pH 7–8.

The excellent water solubility of SHs was related to the release of small hydrophilic peptide fragments at a higher DH from the parent protein by bromelain, which indicates the possibility of its applications as an ingredient in formulated food systems.

In similar research, enzymatic hydrolysis was found to improve the solubility of protein hydrolysates due to the release of soluble peptides from less soluble protein complexes [19].

As previously reported, DH influenced the properties of protein hydrolysates and the foaming properties were found to improve at low DH, possibly due to the inability of the small-sized peptides to stabilize the foam at higher DH [20]. Thus, foam stability of protein hydrolysates is influenced by the molecular weight of the constituent’s peptides [21].

As shown in Figure 4b,c, the pH influenced the foaming capacity and foaming stability of stone fish hydrolysates. The lowest foaming capacity and foaming stability values were obtained at pH 4. However, they both increase to their maximum values at pH 6 and then decrease slightly towards alkaline pH 8 to pH 10.

Therefore, the foaming properties of SHs were greatly affected by the pH of the dispersing medium. An improved foaming property was also related to an increased net charge of protein hydrolysates [22].

The difference between the initial value of foaming capacity and that after 3 min was found to be 75% at pH 2, 50% at pH 4, 25% at pH 6, and 10% at pH 8 and 10. The enhanced foaming stability obtained at higher pH might be attributed to the increased net charge which promoted the intermolecular electrostatic attractions and rigid behaviour of the adsorbed peptides at the air-water interface [22]. Foam stability revealed the extent of peptide-peptide interaction, and its decrease at lower acidic pH might have resulted from the ionic repulsion of the peptide content of the hydrolysates. In similar work, Klompong et al. linked the decreased foam stability of hydrolysates from yellow stripe trevally meat to the shorter peptide chain length, the hydrolysis enzyme used and ionic repulsion between the peptides at extremely acidic pH levels [21]. 

In addition, the water holding capacity of SHs was 5.407 ± 0.001 g/g protein. The water holding capacity (WHC) of protein hydrolysates was reported to be influenced by the protein unfolding and denaturation as well as the appearance of carbohydrates and other non-protein components [18,23]. According to Cumby et al. [24], the water holding capacity obtained for canola protein hydrolysates was affected by the type of hydrolysis enzyme used. Low molecular weight hydrolysates fraction tends to be more hydrophilic with high water-binding capacity. This was attributed to the increased formation of polar groups including –NH_2_ and –COOH groups in the course of hydrolysis [19]. Protein hydrolysates with good water-holding capacity can be incorporated in minced meat to enhance cooking yield [20,24].

Moreover, the fat binding capacity of SHs was 6.25 ± 0.01 mL/g protein. High oil/fat binding capacity is an important quality of protein hydrolysates for use in the confectionery and meat industries. These include the use of hydrolysates to bridge the fat and water content in sausages [25]. The ability of hydrolysates to bind fat/oil can be affected by factors like enzyme–substrate specificity [26], bulk density [23] and degree of hydrolysis [27].

### 2.4. Central Composite Design (CCD) and Model Validation

The results of CCD are given in Table 2. The DH and ACE-inhibitory responses were analyzed by ANOVA using Minitab 16.0 software (MINITAB, State College, PA, USA) to evaluate the significance and adequacy of the second order polynomial model for DH and ACE-inhibitory activity [16].

The individual and combined influence of the independent factors on DH and ACE inhibition were identified using student’s *t* test and *p* values.

The extent of variability in the observed response was measured using the coefficient of determination (R^2^) based on the effects of the process variables and their mode of interactions. The higher R^2^ values obtained indicated a high level of reliability which means the model is well-fitted to the experimental data and that 97.74% and 98.14%, respectively, of the variability in the observed DH and ACE-inhibitory-activity responses can be explained by the model.

The non-significant (*p* > 0.05) lack-of-fit values of 0.076 for DH and 0.771 for the ACE-inhibitory activity further verified the significance of each model and its fitness for the experimental data.

## 3. Materials and Methods

### 3.1. Materials

Fresh samples of stone fish (*A. lecanora*) were locally supplied from Kedah and Langkawi breeding centers in Malaysia. The internal organs were immediately removed, and samples were washed and transported in iced bags to the laboratory. The samples were rewashed, packed in plastic bags and stored at −80 °C until use. Prior to analysis, the samples were freeze dried and grounded with a Waring^®^ blender (Stamford, CT, USA), sieved through 600 µm wire gauze and kept at −40 °C until analysis. Bromelain from pineapple stem tissue, 100 gr (2.4 to 3 U/mg International Pharmaceutical Federation (FIP)) was obtained from Acros Organics (Geel, Belgium). Angiotensin converting enzyme (≥2.0 units/mg protein) and N-Hippuryl-His-Leu hydrate-powder, (≥98%) were purchased from Sigma–Aldrich (St. Louis, MO, USA). Dithiothreitol and *O*-phthaldialdehyde (OPA), 97% were obtained from Sigma–Aldrich (Buchs, Switzerland). All other chemicals used for this study were analytical grade and obtained from Acros organics (Geel, Belgium), Fisher Scientific (Loughborough, Leics, UK), J.T Baker (Bangkok, Thailand) and Merck KGaA (Darmstadt, Germany).

### 3.2. Proximate Composition Analysis

The proximate analysis of the freeze-dried stone fish powder for moisture, ash, crude fat and crude protein were obtained according to the method of the Association of analytical communities AOAC [28]. Total nitrogen content was determined via micro-Kjeldahl method and the crude protein content was calculated as total nitrogen content ×6.25. All determinations were done in triplicate.

### 3.3. Preparation of Stone Fish Hydrolysates with Bromelain

The freeze-dried and powdered sample of stonefish (10 g) was subjected to dialysis for 24 h in a 12–14 kDa molecular mass cut-off dialysis tube (4 h against deionized water at room temperature and 20 h against reaction buffer at 4 °C). After dialysis, the sample was then mixed with 50 mL of the buffer (50 mM, pH 4, 5.5 and 7) under different hydrolysis conditions according to the central composite design using RSM by MINITAB software Version 16.0 (Table 2). The mixture was preheated to the required temperature for each experiment (40 °C, 55 °C and 70 °C). The protease (bromelain) was then added based on the enzyme/substrate ratio as shown in Table 2 and the hydrolysis reaction was carried out at 150 rpm in water bath. The mixture was heated in boiling water at 100 °C for 10 min to inactivate the enzyme and terminate the reaction. After centrifugation for 20 min at 4 °C and 10,000 rpm, the supernatant containing the ACE-inhibitory hydrolysates was collected, freeze dried and placed at −40 °C until use.

### 3.4. Estimation of Protein Concentration

The protein concentration of the stone fish hydrolysates was determined following a modified Lowry method using a micro Bicinchoninic Acid (BCA) protein Assay kit obtained from Sigma–Aldrich.

### 3.5. Determination of Degree of Hydrolysis (DH)

The DH was determined based on OPA spectrophotometric assay according to Nielsen, Petersen, & Dambmann [29] and Li et al. [30] with modification.

The OPA working reagent was freshly prepared as follows: 7.620 g disodium tetraborate decahydrate and 0.20 g sodium dodecyl sulfate were completely dispersed in 150 mL deionized water. 0.160 g *O*-phthaldialdehyde 97% was solubilized in 4 mL ethanol and added to the solution above by rinsing with deionized water. A weight of 0.176 g dithiothreitol (DTT) was dissolved with deionized water and added into the solution above. The final volume of the solution was then adjusted to 200 mL using deionized water. Then, 3 mL of the OPA reagent was added into 400 μL aliquot of stone fish hydrolysates and 400 μL of deionized water (blank), respectively, and gently vortexed for 5 s. The tubes were allowed to stand for 120 s at 25 °C and the absorbance was spectrophotometrically read at 340 nm.

The free amino group content of the samples was calculated as serine amino equivalent from a standard curve constructed using L-serine. The DH of the SHs was then calculated using the following equation:

(1) $$DH\;\left(\%\right)=\frac{L_{t}-L_{0}}{L_{max}-L_{0}}\times 100$$

where *L_t_* is the amount of free amino groups released following hydrolysis at a time *t*, *L*_0_ is the amount of free amino groups in the original stone fish sample before enzyme addition and *L_max_* is the total amount of free amino groups in the original stone fish sample obtained following acid hydrolysis with 6 M HCl at 110 °C for 24 h.

### 3.6. Determination of ACE-Inhibitory Activity

The inhibitory effect of the stone fish hydrolysates on ACE was determined as described by Jimsheena & Gowda [31], with modification. A 15 µL of 1% hydrolysate solution was reacted with 10 µL of ACE (100 mU/mL) and 50 µL of 5 mM Hippuryl-histidyl-leucine (HHL) solution made in 100 mM borate buffer pH 8.3, and 300 mM NaCl. After incubation for 60 min at 37 °C, 75 µL of 1 M HCl was used to terminate the enzymatic reaction followed by the addition of 150 µL of pyridine and 75 µL of benzene sulfonyl chloride. The mixture was vortexed for 1 min and the heat evolved was cooled in ice. Thereafter, 200 µL was added to each well in a 96-well plate and the absorbance at 410 nm was recorded using a microplate reader (Labomed, model UVD-2950, USA Power Wave X 340, Biotek Instruments. Inc., Winooski, VT, USA). One unit of ACE activity was defined as 1 mmol of released hippuric acid (HA) per minute at pH 8.3 and 37 °C:

(2) $$\mathrm{ACE}\;\mathrm{inhibitory}\;\mathrm{activity}\;\left(\%\right)=\frac{\left\lfloor \left(A_{c}-A_{s}\right)\right\rfloor }{\left\lfloor \left(A_{c}-A_{b}\right)\right\rfloor }\text{×}100$$

where *A_s_* is hippuric acid absorbance when ACE, substrate captopril and stone fish hydrolysates are all present; *A_c_* is hippuric acid absorbance when only ACE and substrate captopril are present, and *A_b_* is hippuric acid absorbance when only substrate captopril is present. The percentage for ACE-inhibitory activity was obtained as the mean value of triplicate determinations.

### 3.7. Functional Properties of Stone Fish Protein Hydrolysates Produced under Optimum Hydrolysis Conditions

#### 3.7.1. Solubility Profile

The solubility of SHs was determined in accordance with the method of Tsumura et al. [32], with some modification. Briefly, 1% concentration of SHs was prepared in deionized water and adjusted the pH over a range of values from 2 to 10 with 1 M HCl or 1 M NaOH solution. The solution was stirred at 25 °C for 30 min and centrifuged at 8000× *g* for 10 min. The protein content was then quantified by micro BCA protein assay and expressed as the percentage of total soluble protein according to the following:

(3) $$\mathrm{Solubility}\;\left(\%\right)=\frac{\mathrm{Supernatant}\;\mathrm{content}\;\mathrm{of}\;\mathrm{protein}}{\mathrm{Total}\;\mathrm{content}\;\mathrm{of}\;\mathrm{protein}\;\mathrm{in}\;\mathrm{sample}}\text{×}100$$

#### 3.7.2. Fat Absorption Capacity (FAC)

The ability to absorb fat by SHs was assessed following the description of Shahidi & Han [20], with modification. 500 mg of dried SHs was weighed and mixed in a 50 mL centrifuge tube with 10 mL of corn oil. This was allowed to stand for 30 min at 25 °C with mixing at an interval of 10 min followed by centrifugation at 2000× *g* for 25 min. The supernatant volume was measured and the fat absorption capacity (FAC) was calculated as volume of oil (mL) absorbed per gram of stone fish hydrolysates.

#### 3.7.3. Water Holding Capacity (WHC)

The water-holding capacity was determined as explained by Beuchat [33], with modification. The SHs sample (500 mg) was dissolved in 50 mL of deionized water. After mixing for 120 s, the solution was kept at 25 °C for 30 min and then centrifuged at 5000× *g* for another 30 min. The supernatant was removed and the centrifuge tube was weighed together with the sediment. The WHC was then estimated as grams of water absorbed per gram of stone fish hydrolysates.

#### 3.7.4. Foaming Properties: Foaming Capacity (FC) and Foam Stability (FS)

The foaming capacity (FC) and foam stability (FS) of SHs were assessed according to Sathe & Salunkhe [34] method, with modification. A 0.5% solution of freeze dried SHs was prepared in deionized water. Twenty millilitres of this solution was corrected to pH 2, 4, 6, 8 and 10. The mixture was homogenized at 1600 rpm and 25 °C for 120 s. After whipping, the sample was transferred quickly into a 250 mL measuring glass container and the total volume was noted after 30 s. The foam expansion was then taken as the percentage of volume increase following homogenization. The FC was then determined as follows:

(4) $$\mathrm{FC}\;\left(\%\right)=\frac{V_{f}-V_{i}}{V_{i}}\text{×}100,$$

where *V_f_* is the whipped volume (mL) and *V_i_* the volume (mL) before whipping. The analysis was conducted in triplicate and the FS was determined as the percentage of foam volume that remains after 3 min as shown below:

(5) $$\mathrm{FS}\;\left(\%\right)=\frac{V_{s}-V_{i}}{V_{i}}\text{×}100,$$

where *V_s_* is the volume (mL) after standing and *V_i_* is the volume (mL) before whipping.

### 3.8. Experimental Design and Statistical Analysis

The software Minitab Version 16.0 was used for experimental design, data analysis and model-building. Four process hydrolysis variables, including pH (X1), temperature (X2), E/S ratio (X3) and time (X4), were recruited at three levels for each variable (Table 2). A central composite RSM was used to establish the model by studying the effects of these variables on the response pattern of DH (Y1) and ACE-inhibitory activity (Y2).

The experiments were conducted based on the design presented in Table 2. A total of 31 experimental runs, consisting of 7 central points, 8 axial points and 2^3^ full factorial designs, were generated. Randomization was made to reduce the effects of variation on the experimental responses due to external factors that cannot be explained by the model. The experimental values of the model were fitted using the following second order polynomial equation or its reduced form obtained from the regression coefficient:

(6) $$Y=\beta_{0}+\overset{4}{\underset{i=1}{\sum }}\beta_{i}X_{i}+\overset{4}{\underset{i=1}{\sum }}\beta_{ii}X_{i}^{2}+\overset{4}{\underset{i<j=2}{\sum }}\beta_{ij}X_{i}X_{j},$$

where *Y* is the response or dependent variable (ACE-inhibitory activity and DH) and *β*_0_ is the constant of the model. *β_i_, β_ii_* and *β_ij_* are the model’s regression coefficients. *X_i_* and *X_j_* are the levels of the process or independent variables (*X*_1_, *X*_2_, *X*_3_ and *X*_4_) and represent their linear, quadratic and interaction effects on the responses. The response due to the independent variables was estimated using Minitab software version 16.0. The significance of β-coefficients of each process variable was determined using a student’s *t* test, and ANOVA was applied to assess the significance and fitness of the model, as well as the effects of the process variables on the responses. The accuracy of the reduced final model was further ascertained by comparing the experimental values with the fitted values predicted by the response regression equation.

Three-dimensional (3D) response surface plots were used to show the pattern of relationship between the responses and the levels of each of the process variables being studied. Response optimizer was employed to establish the actual optimum levels of the four process variables that yielded the maximum ACE-inhibitory activity of the stone fish hydrolysates. Under these conditions, confirmatory tests were performed for statistical validation of the experimental procedures.

## 4. Conclusions

In this study, we have successfully established the hydrolysis condition of stone fish with bromelain using RSM. The temperature of 40 °C, E/S ratio at 2%, pH of 7 and time of 4 h were identified as optimal for the hydrolysis of stone fish to generate hydrolysates with strong ACE-inhibitory potential. The experimental data of 84.26% for ACE-inhibitory activity and 44.59% for DH obtained under these conditions were not statistically different from the predicted values of 84.42% and 46.26% for ACE-inhibitory activity and DH as calculated by RSM, within a 95% confidence interval, thus indicating a good fit of the model to the experimental data.

The optimization process involved the whole peptide content of the hydrolysates. This is because it is more efficient, inexpensive, and preserves the nutritional value of the protein. Thus, the ACE-inhibitory effect of the hydrolysates is the net inhibitory effect of the different peptides present.

The stone fish derived ACE-inhibitory hydrolysates are likely to exhibit no life-threatening side effects. Hence, they could have various applications as functional ingredients in the formulation of foods and beverages for the control of blood pressure to reduce the risks of cardiovascular diseases in hypertensive individuals. 

## Figures and Tables

**Table 1:** Proximate composition of freeze-dried stone fish (*Actinopyga lecanora*) sample powder in comparison with other sea cucumber species.

Sea Cucumber Species	Moisture (%)	Ash (%)	Fat (%)	Protein (%)	Carbohydrate (%)	Reference
*Actinopyga lecanora*	5.11 ± 0.142	39.80 ± 0.608	2.03 ± 0.113	45.39 ± 0.044	7.68 ± 0.001	Present study
*Holothuria scabra*	6–6.5	17.91–44.53	1.17–2.44	39.77–60.18	-	[14]
*Holothuria tubulosa*	8.28 ± 0.23	46.43 ± 0.51	0.71 ± 0.12	44.58 ± 1.01	-	[15]
*Holothuria polii*	10.23 ± 1.03	48.22 ± 1.02	0.55 ± 0.12	36.99 ± 0.62	-	[15]

All the values are expressed as mean ± standard deviation (SD).

**Table 2:** Central composite experimental design, predicted and response values of the two dependent variables under different reaction conditions.

Run Order	X1	X2	X3	X4	% Degree of Hydrolysis (Y1)		% ACE Inhibition (Y2)	
					Predicted	Experimental	Predicted	Experimental
1	4	70	2	30	36.34	34.49	63.88	64.70
2	5.5	55	1.25	195	54.64	56.66	71.82	68.24
3	5.5	55	0.5	195	43.68	42.10	63.46	64.86
4	4	70	0.5	360	31.17	29.96	59.71	59.66
5	5.5	55	1.25	360	55.52	57.82	66.34	65.49
6	7	55	1.25	195	44.19	43.00	69.18	70.95
7	7	40	0.5	360	32.41	32.25	50.37	51.35
8	7	40	0.5	30	24.35	23.96	43.51	42.81
9	4	70	0.5	30	28.03	28.25	60.53	60.92
10	4	40	2	360	53.34	51.99	79.61	79.55
11	5.5	55	1.25	195	54.64	54.81	71.82	72.30
12	7	70	0.5	30	28.43	29.25	49.12	49.31
13	4	40	0.5	360	32.00	31.89	73.54	72.73
14	5.5	40	1.25	195	53.83	51.86	74.56	72.83
15	5.5	70	1.25	195	55.45	57.56	69.08	68.95
16	5.5	55	1.25	195	54.64	53.56	71.82	72.30
17	5.5	55	2	195	56.20	58.64	80.18	80.05
18	5.5	55	1.25	195	54.64	53.95	71.82	72.30
19	5.5	55	1.25	195	54.64	54.05	71.82	72.16
20	7	40	2	360	49.13	50.62	80.44	81.16
21	5.5	55	1.25	30	43.41	41.98	63.32	61.27
22	4	40	0.5	30	23.95	25.03	66.69	67.96
23	7	70	0.5	360	31.57	32.90	48.30	47.71
24	4	70	2	360	52.50	51.25	63.06	62.68
25	7	40	2	30	28.06	26.69	73.58	73.24
26	7	70	2	30	32.14	32.65	76.48	75.70
27	4	40	2	30	32.26	34.68	72.75	73.94
28	4	55	1.25	195	46.09	48.14	74.46	74.40
29	5.5	55	1.25	195	54.64	53.82	71.82	72.16
30	5.5	55	1.25	195	54.64	53.79	71.82	72.16
31	7	70	2	360	48.30	47.25	75.66	76.70

Process variables: X1, hydrolysis pH; X2, hydrolysis temperature °C, X3, hydrolysis E/S ratio (%) and X4, hydrolysis time (min). Response variables; Y1, degree of hydrolysis (DH, %) and Y2, ACE-inhibitory activity (%). ACE; angiotensin I-converting enzyme.

**Table 3:** Analysis of variance and estimated regression coefficients for DH (Y1) and ACE-inhibitory activity (Y2) quadratic models.

Source	Coefficients		F		*t*		P	
	Y1	Y2	Y1	Y2	Y1	Y2	Y1	Y2
Regression			131.01	176.83			0.000	0.000
Linear			105.89	138.63			0.000	0.000
Squares			260.02	216.90			0.000	0.000
Interaction			23.63	114.74			0.000	0.000
Lack-of-Fit							0.076	0.771
**Terms**								
Constant	−116.530	129.843			−9.149	22.841	0.000	0.000
X1	47.132	−15.625			9.284	−17.372	0.000	0.000
X2	0.151	−0.675			3.439	−7.217	0.003	0.000
X3	29.774	−14.882			5.801	−6.493	0.000	0.000
X4	0.105	0.152			5.730	15.029	0.000	0.000
X1·X1	−4.226				−9.213		0.000	
X3·X3	−8.371				−4.562		0.000	
X4·X4	−0.000	−0.000			−5.017	−14.728	0.000	0.000
X1·X2		0.131				9.023		0.000
X1·X3	−1.024	5.335			−2.646	18.400	0.016	0.000
X2·X3		−0.060				−2.081		0.050
X2·X4	−0.000	−0.001			−2.824	−5.885	0.010	0.000
X3·X4	0.026				7.477		0.000	
Y1; SD = 1.74, R^2^ = 98.50%, R^2^ (adj) = 97.74%								
Y2; SD = 1.30, R^2^ = 98.70%, R^2^ (adj) = 98.14%								

F = Fisher, *t* = Student’s *t* test, P = probability, X1 = hydrolysis pH, X2 = hydrolysis temperature (°C), X3 = hydrolysis E/S ratio (%), X4 = hydrolysis time (min), SD = standard deviation, R^2^ = R squared, R^2^ (adj) = adjusted R squared.

## Abbreviations

ACE	Angiotensin converting enzyme
HHL	Hippuryl-histidyl-leucine
DH	Degree of hydrolysis
OPA	*O*-phthaldialdehyde
RSM	Response surface methodology
SHs	Stone fish hydrolysates
CCD	Central composite design
BCA	Bicinchoninic Acid
DTT	Dithiothreitol
WHC	Water Holding Capacity
FAC	Fat Absorption Capacity
FC	Foaming Capacity
FS	Foam Stability
FIP	International Pharmaceutical Federation
AOAC	Association of Analytical Communities
